# Effects of the areas of worklife on job embeddedness: a national cross-sectional study among Egyptian nurses

**DOI:** 10.1186/s12912-022-01107-6

**Published:** 2022-12-12

**Authors:** Heba E. El-Gazar, Shymaa Abdelhafez, Mohamed A Zoromba

**Affiliations:** 1 Nursing Administration Department, Faculty of Nursing, Port Said University, Port Said, Egypt; 2 Department of Nursing, College of Applied Medical Sciences, Prince Sattam Bin Abdulaziz University, Al-Kharj, Saudi Arabia; 3 Psychiatric and Mental Health Nursing Department, Faculty of Nursing, Mansoura University, El-Mansoura, Egypt

**Keywords:** Areas of work life, Job embeddedness, Nurses, Person–job match

## Abstract

**Aim:**

This study aimed to examine the effects of a person–job match in the six areas of worklife on Egyptian nurses’ job embeddedness.

**Background:**

Healthcare organizations struggle to embed nurses in their job. However, the antecedents of nurses’ job embeddedness are not fully known, especially those related to organizational factors. This study is an initiative to contribute in this field.

**Methods:**

A national cross-sectional study that enrolled 1003 Egyptian licensed nurses was conducted. Data were collected using an online-based version of the Areas of Worklife Scale and the Global Job Embeddedness Scale and analyzed using the descriptive statistics, Pearson correlation, and regression analysis.

**Results:**

Nurses reported a moderate level of job embeddedness with a person–job match in control, reward, and community. Regression analysis showed four of six areas of worklife (value, fairness, community, and control) contributing to nurses’ job embeddedness.

**Conclusion:**

Nurses who experience a person–job match in the value, fairness, community, and control areas of worklife are more likely to embed in their job. A match in the value area has the great potentials for nurses to embed in their job.

**Implications for nursing management:**

Actions aimed at embedding nurses should prioritize on optimizing a person–job match in value, fairness, community, and control.

## Introduction

Nursing, which is the backbone of healthcare organizations and the first contact point with the patients [1], suffers from an increasing turnover rate worldwide [2]. The global nurse turnover rate ranged between 15.1% and 44.3% [3]. In Egypt, the problem is further worsening considering the high shortage (19.3 nurses/10,000 population) more than the global range [4] and high level of Egyptian nurse migration [5]. Losing nurses is costly because of new nurses’ orientation, which is estimated to be approximately 1.3 times their annual salary. Moreover, it has an indirect effect, resulting from the shortage of nurses, which negatively affects productivity, patients’ safety, nursing care quality, and nurse satisfaction [6]. These deleterious effects urge researchers and practitioners for several years to search for what makes nurses leave their job, but unfortunately, they have not yet fully identified solutions to effectively retain nurses [7].

In this backdrop, job embeddedness has emerged as an anti-withdrawal means investigate the collection of forces which keep nurses stay in their job [8, 9]. Since the initial conceptualization of job embeddedness, scholars have become increasingly interested in it and call for studies that gain in-depth knowledge and better understanding regarding the predicators of nurses’ job embeddedness [10–13]. In responding to this call, a previous research studied job embeddedness predictors and argued that nursing leadership styles, such as humble [14] and authentic leadership [15] styles, can predict nurses’ job embeddedness. Another study proposed that nurses’ self-efficacy may be an antecedence to job embeddedness [16].

Despite the finding that a suitable work environment may foster nurses’ retention [17, 18], there is little insight in the literature on the relationship between the work environment and nurses’ job embeddedness [10, 14]. One of the promising ways to examine work environment is the areas of work life, which investigate the person–job match within six domains: workload, control, reward, community, fairness, and value [19]. The link between the areas of worklife and nurses’ job embeddedness was not studied before, and to the best of the authors’ knowledge, this study is an initiative to fill this gap. Therefore, this study aimed to explore the effects of the person–job match in the six areas of worklife on Egyptian nurses’ job embeddedness.

## Background

The six areas of worklife were theorized by Maslach and Leiter to examine the differences between one’s job expectations and their actual working conditions (i.e., person–job match) [20]. The six areas involve “manageable workload,” which reflects the job demand in relation to available time and resources; “control,” which means the professional autonomy that enables one to choose and access the necessary resources to perform their job effectively; “reward,” which refers to financial, social, and/or internal recognition for one’s contributions to work; “community,” which includes the quality of working relationships, sense of closeness, humor, and social support from managers, colleagues, and subordinates; “fairness,” which represent openness, respect, and equity of workload, resource, and reward; and “value,” which means the extent to which the organization’s priorities, goals, and ethics matched with those of the staff. Achieving a high degree in these areas indicate a person–job match [21].

Maslach and Leiter’s theory of person–job match within the six areas of worklife has suggested a model linking this theory with job burnout [21]. This model proposes that the mismatch between the person and the job in some or all six areas of worklife results in burnout. On the other hand, the greater the match, the greater the likelihood of involvement, energy, and positive efficacy. Subsequent studies have confirmed that the degree of fit between the person and the job within the six areas of worklife determines the degree to which a person experiences burnout [22–24].

Further, a plethora of research assured the vital role of the person–job match in six areas of worklife among nurses in gaining positive work-related outcomes. When nurses perceive a person–job match, they showed more work engagement [25] and civility behaviors and less emotional exhaustion [23]. In addition, person–job mismatch among nurses can affect their physical and mental health such as fatigue, depressive symptoms, sleep difficulties, shortness of breath, and muscle trembling [26]. According to Boamah and Laschinger, achieving a person–job match in the areas of worklife could be used as a strategy to enhance nurses’ retention [18]. The recent construct used in the literature to describe nurses’ retention is job embeddedness [9, 10].

Job embeddedness is the extent to which nurses are enmeshed, attached, and tied to the healthcare organization where they work [9]. The notion of job embeddedness differs from the traditional turnover model in that job embeddedness focuses on the causes that make nurses remain in the current job, rather than on the causes that make nurses leave [27]. It also differs from the traditional retention model in that it expands the retention model to consider the importance of both nurses’ attachment to organization and community [7].

The original theory of job embeddedness conceptualizes that job embeddedness consists of two components: on-the-job embeddedness (person’s tie to one’s organization) and off-the-job embeddedness (person’s tie to one’s community). Each of these components has three critical aspects: (a) link, the connections between the person and organizations, activities, and other people, either formal or informal; (b) fit, compatibility and comfort a person feels with their organization; and (c) sacrifice, giving up valued things if the person decides to leave their job [8]. All of these components were united in a single dimension construct by Crossley et al. who proposed a global measure of job embeddedness [28].

Worldwide, healthcare organizations struggle to retain and embed nurses in their job [13, 17]. Highly embedded nurses are more likely to show a high level of job performance, innovation, citizenship behavior, commitment, and organizational engagement [11]. Moreover, job embeddedness increases the attachment and retention of nurses in their hospitals [14], which makes it the solution to nurse shortages and turnover [7]. This highlights the need to examine the levels of job embeddedness among nurses, particularly in countries with high nursing shortages, such as Egypt. A better insight into the nurses’ job embeddedness levels can help hospital administrators implement proactive measures to enhance them, preventing the potential harm resulting from inadequate nurses’ job embeddedness. However, very few studies have been conducted to explore the levels of job embeddedness among Egyptian nurses [29, 30].

This study aimed to investigate person–job match/mismatch in worklife among Egyptian nurses. Unfit between the person and his job in one or more areas of worklife is associated with deleterious individual and organizational outcomes, such as poor care quality, increased turnover, low productivity, and absenteeism [18]. These effects have sustained a continuing interest in assessing person–job match/mismatch within the six areas of work-life. Nevertheless, there is no published study exploring person–job match/mismatch within Egyptian nurses’ six areas of work-life.

Further, in light of previous mentioned importance of nurses’ job embeddedness and given that the factors that prevent nurses to embed and stay in their job are unknown, there is a growing need to identify the predicators of nurses’ job embeddedness [11], particularly those related to nurse work environment [17]. This study is an initiative to contribute to job embeddedness literature by integrating the concepts of the areas of the worklife by Maslach and Leiter and the theory of job embeddedness by Mitchell et al. [8, 20]. The contribution of these studies could help nurse managers and hospital administrators develop appropriate strategies to enhance nurses’ job embeddedness, which finally lead to retain qualified nurses.

### Aim

This study aimed to explore the effects of the person–job match in the six areas of worklife on Egyptian nurses’ job embeddedness.

This study sought to answer the following research questions:

- In Egyptian nurses, what is the job embeddedness level?
- In Egyptian nurses, what are areas of worklife they experience in a person–job match/mismatch?
- In Egyptian nurses, what are the areas of worklife that predict nurses’ job embeddedness?

#### Subjects and methods

##### Study design

A descriptive cross-sectional online survey was conducted among Egyptian nurses.

##### Participants

Convenience sampling was used to recruit participants online who were licensed nurses (staff nurses and nursing leaders), worked in secondary (300–500 beds) or tertiary (over 500 beds) Egyptian hospitals for at least 1 year, and were on duty during the time of data collection. Nurses who were interns or on vacation or left the nursing profession or worked in primary healthcare facilities (health centers or clinics) were excluded.

The required sample size was calculated via power analysis using G*Power software version 3.1.9.4 for regression analysis [31] with an 80% power, an alpha set at 0.05, a small effect size set at 0.02 [32], and 14 predicators (six dimensions of the areas of worklife and eight demographics), and a sample size over 929 nurses was needed to be approached. A total of 1,003 nurses from 53 hospitals (governmental = 34, educational = 12, and private = 7) completed the online survey.

##### Measures

The Areas of Worklife Scale (AWS); the Global Job Embeddedness Scale (JES), developed in English; and a demographic form were used in this study. Both the English scales followed the Beaton et al. standard for the translation process [33]. First, each English scale was translated from English to Arabic by the first author and then back-translated from Arabic to English by a qualified translator. Next, the translated and back-translated versions were compared against the original English scale. Corrections were made, and the translated version matched closely with the original English version. Subsequently, a panel of experts composed of three nursing researchers, two nursing directors, and two nursing specialists reviewed the translated version against the original version to ensure equivalence of terms and verify that the language is clear and simple. Some words were modified to better fit the context of Egyptian nurses. Finally, the translated scales were pilot tested among 36 nurses, enrolled online, who verified the final version. Cronbach’s alpha for the AWS in the pilot study ranged from 0.627 to 0.809, and it was 0.825 in the Global JES. The responses of both scales were measured along a 5-point Likert scale ranging from 1 (strongly disagree) to 5 (strongly agree).

### AWS

This scale was developed by Leiter and Maslach [21] and adopted from Fernando-Gertum [34]. It consists of 29 items examining the person–job match or mismatch in the six areas of work life: manageable workload (six items), control (three items), reward (four items), community (five items), fairness (six items), and value (five items). Each area includes positively worded items of matching (e.g., “Members of my workgroup cooperate with one another” [community]) and negatively worded items of mismatching (e.g., “My efforts usually go unnoticed” [reward]). The negatively worded items are scored in reverse. The scale produces distinct scores for each area. A score greater than 3.0 indicates that nurses experience a match with their working conditions. Cronbach’s alpha for the scale in its original version ranged from 0.70 to 0.82, and in this study, it ranged from 0.66 to 0.90.

The confirmatory factor analysis (CFA) in this study was applied by testing two models. The first model loaded all the scale items onto a single-factor model. The second model tested the proposed six-factor structure. The results showed a better fit of the six-factor structure than a one-factor model (χ2 = 2059.360, df = 327, χ2/df = 6.298, CFI = 0.909, TLI = 0.901, RMSEA = 0.073 vs. χ2 = 5958.584, df = 377, χ2/df = 15.805, CFI = 0.708, TLI = 0.685, RMSEA = 0.122).

### The Global JES

This scale was developed by Crossley et al. [28]. It consists of seven items with a single dimension, representing nurses’ general level of embeddedness in their job. A sample item is “I feel attached to this hospital.” Item 6 is scored in reverse. The higher the score, the higher the degree of nurses’ job embeddedness. Cronbach’s alpha for the scale in its original version was 0.88, and it was 0.90 in this study. The results of the CFA in this study showed a satisfactory fit with the data (χ2 = 55.476, df = 12, χ2/df = 4.623, CFI = 0.990, TLI = 0.982, RMSEA = 0.060).

### The demographic form

The demographic form includes information about age, sex, marital status, educational degree, position, hospital type, years in nursing, and years in the current hospital.

#### Data collection and ethical consideration

Data were collected using a Web-based survey, generated by Google Forms, a free online survey platform. The survey link accompanied with an invitation was shared to nurses on Facebook pages for nurses in Egypt and through private messages on Facebook Messenger and WhatsApp platforms. Data regarding who should participate in the study and the study purpose were posted in the invitation letter. Confidentiality and voluntary participation were assured. An online informed consent form was developed, and agreeing on it, through a click on an “I agree” button, was set as a condition that the participants could proceed to the survey questions. All survey questions were set as required questions that could not be possibly submitted with incomplete answers. Participants were not permitted to submit more than one response from the same account using the same device. Once the number of responses exceeded the calculated sample size, the researchers shut down the survey page. Data were collected during January and March 2021.

#### Statistical analysis

Statistical analysis was performed using SPSS software version 24 (IBM Corporation, Chicago, IL, USA) and Amos version 23.0. Numbers and percentages were used to quantify the participated nurses’ demographic characteristics. The construct validity of each measure used in this study was assessed using CFA. Cronbach’s alpha was applied to assess the internal consistency of the study scales, which needed to be at least 0.5 and preferably over 0.7 [35]. Means and standard deviations were computed for the study variables. An independent sample t-test and one-way analysis of variance (ANOVA) were used to identify differences in the study outcomes according to demographic characteristics. The Pearson r correlation coefficient was used to determine the relationships between the six areas of worklife and job embeddedness. Due to the study aim is a prediction of job embeddedness, not verification from a hypothesized model, regression analysis is more suitable to achieve the study aim. Stepwise multiple regression analyses were computed between job embeddedness and variables that yielded a significance in difference and correlation tests. The lack of multicollinearity was assured, indicated by the tolerance (0.46–0.63 (> 0.1)) and variance inflation factor (1.58–2.17 (< 3)). *P*-value < 0.05 considered statistically significant.

#### Common method bias (CMB)

The data in this study were collected from a single source using self-reported measures, which increased the risk of CMB. To address this concern, several procedural remedies and statistical remedies were applied. The procedural remedies were applied by providing the participants with detailed information on the purpose of the study, guaranteeing voluntary participation, anonymity, and confidentiality, and improving the scale items’ clarity by pilot testing within the target population [36]. The statistical remedies included Harman’s single-factor test to assess the presence of CMB [37], which showed that the single factor demonstrated 41.622% of the variance, below the cut–off point of 50% [38]. This indicated that CMV was not a major issue in this study. Furthermore, the results of the CFA of the AWS scale, which revealed a better fit of the multi-dimension model than the single-factor model, were evidence that the data had no method of common bias deviation.

## Results

### Preliminary analysis

The sample participants were mostly female (80.6%) and working in governmental hospitals (71.4%) as staff nurses (78.4%). Moreover, 39.8% of them were aged between 30 and 40 years, and 32.0% had a nursing diploma degree. Regarding marital status, 63.5% of the participants were married, and 36.5% were unmarried (including those who were single, divorced, or widowed). As for their work experience, less than half of the participants had spent between 10 and 20 years in nursing profession (43.4%) and less than 5 years in their current hospital (42.9%). There was no statistically significant difference in job embeddedness according to nurses’ demographics (Table 1).

**Table 1:** Participants’ demographics and differences in job embeddedness (*N* = 1,003)

Characteristic	Category	No	%	Job embeddedness	
				M (SD)	t/F (P)
**Age (years)**	< 30	347	34.6	2.92 (0.80)	*F* = 1.16 (0.31)
	30–40	399	39.8	2.84 (0.80)	
	> 40	257	25.6	2.92 (0.877)	
**Sex**	Male	195	19.4	2.93 (0.85)	*t* = 0.77 (0.44)
	Female	808	80.6	2.88 (0.78)	
**Marital status**	Married	637	63.5	2.89 (0.79)	*t* = 0.43 (0.67)
	Unmarried	366	36.5	2.87 (0.80)	
**Education**	Diploma	321	32.0	2.86 (0.78)	*F* = 1.21 (0.31)
	Associate	290	28.9	2.96 (0.79)	
	Bachelor	301	30.0	2.87 (083)	
	Postgraduate	91	9.1	2.82 (0.73)	
**Position**	Staff nurse	786	78.4	2.88 (0.81)	*t* = − 0.63 (0.53)
	Nurse manager	217	21.6	2.92 (0.74)	
**Hospital type**	Governmental	716	71.4	2.88 (0.78)	*F* = 0.06 (0.95)
	Private	139	13.8	2.89 (0.79)	
	Educational	148	14.8	2.90 (0.84)	
**Nursing tenure (years)**	< 10	356	35.5	2.86 (0.82)	*F* = 0.39 (0.67)
	10–20	435	43.4	2.89 (0.79)	
	> 20	212	21.1	2.92 (0.76)	
**Hospital tenure (years)**	< 5	430	42.9	2.89 (0.83)	*F* = 0.55 (0.58)
	5–10	357	35.6	2.86 (0.77)	
	> 10	216	21.5	2.94 (0.75)	

As shown in Table 2, the nurse participants reported a person–job match in control, community, and reward (M = 3.12, SD = 1.08; M = 3.04, SD = 0.75; and M = 3.02, SD = 0.49, respectively). Meanwhile, nurses reported a person–job mismatch in workload, fairness, and value (M = 2.74, SD = 0. 69; M = 2.86, SD = 0. 52; and M = 2.98, SD = 0.75, respectively). The mean score of nurses’ job embeddedness was 3.36 (SD = 0.9) of 5, indicating a moderate level of nurses’ embeddedness in their job. There was a significant positive relationship between all six areas of worklife and job embeddedness among Egyptian nurses.

**Table 2:** Cronbach’s alphas, means, standard deviations, and correlations (*N* = 1,003)

Variable	α	Mean ± SD	1	2	3	4	5	6	7
1. Workload	0.66	2.74 ± 0.69	1	0.38**	0.19**	0.45**	0.32**	0.33**	0.34**
2. Control	0.80	3.12 ± 1.08		1	0.19**	0.58**	0.40**	0.54**	0.51**
3. Reward	0.84	3.02 ± 0.49			1	0.27**	0.23**	0.19**	0.21**
4. Community	0.90	3.04 ± 0.75				1	0.57**	0.64**	0.61**
5. Fairness	0.88	2.86 ± 0.52					1	0.51**	0.55**
6. Value	0.82	2.98 ± 0.75						1	0.69**
7. Job embeddedness	0.90	2.89 ± 0.79							1

**P < 0.01

### Regression analysis

A stepwise multiple regression analysis was performed to further explore the relationship between nurses’ job embeddedness as a dependent variable and person–job match in the six areas of worklife (manageable workload, control, reward, community, fairness, and value) as predicators, which were statistically significant with job embeddedness. None of the sociodemographic variables were entered into the multiple regression equation as there was no significant difference in the job embeddedness variable (Table 1).

Four of the six areas of worklife (value, fairness, community, and control) were found to be significant predicators and explained 56.0% of the total variance in nurses’ job embeddedness (*F* = 317.54, *P* = 0.000). Workload (*t* = 1.17, *p* = 0.24, β = 0.03) and reward (*t* = 0.68, *p* = 0.49, β = 0.02) were excluded. In addition, the regression coefficient revealed that nurses’ job embeddedness was mostly affected by value, followed by fairness, community, and control (β = 0.44, 0.19, 0.17, and 0.09, respectively; Table 3).

**Table 3:** Results of a stepwise multiple linear regression analysis predicting job embeddedness of the studied nurses (*N* = 1,003)

Predicators	B	SE (B)	β	*T*	*P* value	95% CI
Constant	−0.065	0.095	—	−0.684	0.494	−0.253–0.122
Value	0.46	0.03	0.44	15.19	< 0.001	0.404–0.524
Fairness	0.28	0.04	0.19	7.08	< 0.001	0.205–0.361
Community	0.18	0.03	0.17	5.55	< 0.001	0.117–0.245
Control	0.07	0.02	0.09	3.42	0.001	0.029–0.105

*R* = 0.748; *R*^2^ = 0.560; Δ*R*^2^ = 0.558; *F* = 317, 54, 54;

*SE* standard error, *β* standardized regression coefficient, *95% CI* 95% confidence interval

*P* < 0.001

## Discussion

This study aimed to examine the effects of the person–job match in the six areas of worklife on Egyptian nurses’ job embeddedness. The results indicated a moderate level of job embeddedness among a sample of Egyptian nurses, showing that they had a good attachment to their organization. This result is similar to those found in Northern Cyprus [12] and Pakistan [39]. Meanwhile, it was higher than those reported in England [40] and lower than those reported in Thailand [27] and the United States [13]. The discrepancy in findings may be related to different work climate, cultural and socioeconomic variations, and the difference in the characteristics of studied nurses. Further researches need to be conducted to identify the factors causing this difference.

Job embeddedness is a construct related to culture and context [28], and Egyptian nurses work in a supportive atmosphere [41], which may explain the results. Although this result is somewhat satisfactory, it raises a concern. As job embeddedness represents an effective strategy, hospital administrators can use it to achieve nurse retention and compensate for the severe nursing shortage [7]. The higher level of nurses’ job embeddedness is related to positive work outcomes [11]. Therefore, enhancing it is not an option, and remarkable efforts are required to promote the level of nurses’ job embeddedness.

Nurses included in this study reported a person–job match in control, reward, and community with the highest match in the control area. These significant findings indicated the nature of the work climate in the study settings that permitted nurses to choose and acknowledge their contributions and facilitated a supportive work environment. These findings are in line with those reported from New Jersey, United States, which included school nurses [42], and those from Italy, which included nurses and other healthcare workers [25]. Meanwhile, other studies from Canada showed a match in the same areas, but the highest match was reported in the community [18] or reward [15] area. Reporting matches in these areas might be associated with their personalities, culture, traditions, and social nature. In support of this explanation, a previous study has asserted that Egyptian nurses work with humble leaders who appreciate their efforts and make them feel empowered [43].

Furthermore, nurses in the current sample reported a person–job mismatch in workload, fairness, and value. International studies also showed a mismatch of nurses’ expectation in the areas of workload and fairness but showed a match in the value area [15, 44, 45]. Of note, the manageable workload area was rated the least from nurses’ expectation, which was consistent with findings of Wong et al. [14]. The high mismatch in workload might be due to a severe shortage of nurses in Egypt and the heavy duties and responsibilities they had after the COVID-19 outbreak. Addressing each area of worklife for nurses is crucial to feel that they are valued [23]. Therefore, in light of these results, nurse managers should discuss with nurses what tasks they find unmanageable and practices they perceive unfair and that did not match with their values and work together to find ways to address these issues.

In terms of job embeddedness predicators, four areas of worklife (control, community, fairness, and value) are considered the strong predicators of nurses’ job embeddedness in this study. These results are consistent with Boamah and Laschinger in that these areas are significant factors influencing nurses’ intention to stay in their jobs [18] and partially support models proposed that control, fairness, and value congruence decrease nurses’ intention to leave the work [46]. This was probably because when nurses found that their organization considered their efforts, had good relations with them, treated them fairly, and aligned with their values, they believed they were members of the organization and were more likely to feel they belonged to the organization. These findings are significant, especially in light of current challenges facing the nursing profession: shortage, high rates of absenteeism, job dissatisfaction, and drop in nurses’ recruitment and retention [47]. Nurse managers could use these results to enhance embeddedness of nurses in their job.

A match in the value area holds the strongest predictor of nurses’ job embeddedness in the current study. That is, when nurses’ values mismatched those of the hospital they worked, this may increase moral distress and discrepancy [48], which in turn may lead to low job embeddedness. In line with that, Leiter and Maslach confirmed that values are the ideals that attract individuals to their job [21]. In addition, earlier studies found that the involvement of healthcare workers was predominantly predicted by value congruence [25, 49]. However, nurses reported a mismatch in the value area, which is alarming. Accordingly, such result could help identify interventions in priority to increase nurse embeddedness in their job.

Despite the significant relationship between manageable workload, reward, and nurses’ job embeddedness, the effects of manageable workload and reward on nurses’ job embeddedness were found to be insignificant. These results are unexpected and contradictory to what is repeatedly known that manageable workload and reward are the leading factors that increase nurses’ retention [18, 46, 50]. However, these findings can be attributed to nurses’ sense of ability to make a choice in their work, free from policy constrains and perceive equity and making a good relationship with work colleagues and supervisors who make nurses feel comfortable and adhere to their job despite workload and the lack of reward. Supporting this notion, Danhakl et al. emphasized that work hours are not the largest contributor to workload stress and the largest sources of workload stress pertain to the lack of mentorship [51]. In addition, Gregory and Menser stated that burnout occurs because of the relationships individuals have in the work and the nature of the workplace rather than the rewards of performing the work [52].

### Implications for nursing management

The study revealed the current level of job embeddedness among Egyptian nurses. These findings can be valuable for nurse managers and hospital administrators to identify the current state of nurses’ embeddedness in their job. By determining the actual level of nurses’ job embeddedness, they can deal with it adequately and make the right decisions to promote it. The study also showed areas of worklife the Egyptian nurses experienced in a person–job match/mismatch. These findings can add to practice and enrich the existing literature. Using these findings, hospital administrators can determine areas where they have succeeded in fulfilling nurses’ expectations and areas that still need further enhancements.

In addition, the current study provided insights into the factors predicating nurses’ job embeddedness, which may have significant practical implications for both nursing managers and human resources managers. The study results highlighted the need for establishing work environment that promotes a person–job match especially in the areas of value, fairness, community, and control to embed nurses in their jobs. Such work environment could be demonstrated in practice by aligning organizational value priorities and goals with those of the nurses, treating them fairly, encouraging a supportive workplace, and involving them in decisions concerning them. This study did not show the perfect everyday practice but at least provided practices that fit with nurses’ expectation.

Moreover, this study helps nursing managers to identify the area in priority to target for interventions, which is the value area. This study also provides empirical evidence that nurses who experience a person–job match in value, fairness, community, and control may be embedded in their job compared with those who experience a match in workload and reward. These findings could shift the way nurse managers think about the natural of nurses and provide them with new insights when managing their staff. This study also contributes to the literature by extensively determining nurses’ job embeddedness.

## Conclusion

Optimizing a person–job match in the worklife areas, namely, value, fairness, community, and control, had the potential to maximize nurses’ job embeddedness. When nurses work in an environment with values, goals, and priorities that align with those of nurses and match their expectation in dealing with equity and respecting and promoting supportive workplace and professional autonomy, they are more likely to embed in their jobs, and withdrawal would be difficult. A match in the value area holds the strongest potential for nurses to embed in their job. A person–job match in workload and reward did not predict nurses’ job embeddedness.

### Limitations and future research directions

This study has some limitations. First, a convenience sample was used, which may have restricted the generalizability of these findings. Randomized samples are recommended in future research. Another limitation is the study utilized a cross-sectional design which was insufficient for determining causal relationships between the study variables. Further longitudinal designs are recommended to confirm these relationships. In addition, this study was conducted among Egyptian nurses, which increases the possibility that the findings were affected by cultural and national influence. Similar studies in different cultural contexts are required to validate the study findings. Finally, this study investigated only six areas of worklife as predicators of nurses’ job embeddedness. Further research exploring other predicators such as workplace cohesiveness, job stress, and conflict strategy used is needed to provide more evidence of the type of intervention nurse managers could implement to enforce nurses to embed in their job.

## Data Availability

The datasets generated during and analyzed during the current study are not publicly available due to confidentiality agreements, but are available upon reasonable request from the corresponding author.

## Abbreviations

AWS: The Areas of Worklife Scale
JES: Job Embeddedness Scale
CFA: Confirmatory factor analysis
CMB: Common method bias
